# Population genetic structure of the malaria vector *Anopheles nili *in sub-Saharan Africa

**DOI:** 10.1186/1475-2875-9-161

**Published:** 2010-06-12

**Authors:** Cyrille Ndo, Christophe Antonio-Nkondjio, Anna Cohuet, Diego Ayala, Pierre Kengne, Isabelle Morlais, Parfait H Awono-Ambene, Daniel Couret, Pierre Ngassam, Didier Fontenille, Frédéric Simard

**Affiliations:** 1 Laboratoire de Recherche sur le Paludisme, Organisation de Coordination pour la lutte Contre les Endémies en Afrique Centrale (OCEAC), P.O. Box 288, Yaoundé, Cameroon; 2 Faculty of Sciences, University of Yaoundé I, P.O. Box 812, Yaoundé, Cameroon; 3 Institut de Recherche pour le Développement (IRD), UR 016, 911 Avenue Agropolis, P.O. Box 64501, 34394 Montpellier Cedex 5, France; 4 Institut de Recherche en Sciences de la Santé (IRSS), P.O. Box 545, Bobo-Dioulasso, Burkina Faso

## Abstract

**Background:**

*Anopheles nili *is a widespread efficient vector of human malaria parasites in the humid savannas and forested areas of sub-Saharan Africa. Understanding *An. nili *population structure and gene flow patterns could be useful for the development of locally-adapted vector control measures.

**Methods:**

Polymorphism at eleven recently developed microsatelitte markers, and sequence variation in four genes within the 28s rDNA subunit (ITS2 and D3) and mtDNA (COII and ND4) were assessed to explore the level of genetic variability and differentiation among nine populations of *An. nili *from Senegal, Ivory Coast, Burkina Faso, Nigeria, Cameroon and the Democratic Republic of Congo (DRC).

**Results:**

All microsatellite loci successfully amplified in all populations, showing high and very similar levels of genetic diversity in populations from West Africa and Cameroon (mean Rs = 8.10-8.88, mean He = 0.805-0.849) and much lower diversity in the Kenge population from DRC (mean Rs = 5.43, mean He = 0.594). Bayesian clustering analysis of microsatellite allelic frequencies revealed two main genetic clusters in the dataset. The first one included only the Kenge population and the second grouped together all other populations. High Fst estimates based on microsatellites (Fst > 0.118, P < 0.001) were observed in all comparisons between Kenge and all other populations. By contrast, low Fst estimates (Fst < 0.022, P < 0.05) were observed between populations within the second cluster. The correlation between genetic and geographic distances was weak and possibly obscured by demographic instability. Sequence variation in mtDNA genes matched these results, whereas low polymorphism in rDNA genes prevented detection of any population substructure at this geographical scale.

**Conclusion:**

Overall, high genetic homogeneity of the *An. nili *gene pool was found across its distribution range in West and Central Africa, although demographic events probably resulted in a higher level of genetic isolation in the marginal population of Kenge (DRC). The role of the equatorial forest block as a barrier to gene flow and the implication of such findings for vector control are discussed.

## Background

The recent shift in strategic emphasis from malaria control to elimination and eradication has highlighted major gaps in knowledge that need to be addressed before such achievement is contemplated [1-3]. Vector control is a cornerstone of malaria prevention strategies and it has been widely acknowledged that renewed efforts in this field should be considered a central aspect of the new malaria eradication strategy [4-6]. Basic knowledge in mosquito vectors biology, ecology and genetics is crucially needed for the development of innovative, integrated and biologically lucid vector management strategies. This is especially true in the malaria heartlands of sub-Saharan Africa, where a number of mosquito species efficiently transmit malaria to humans simultaneously, or replace each other seasonally sustaining year-round transmission [7-9]. Any strategy aiming at reducing transmission down to the level where elimination is within reach will need to transcend the relevant biodiversity of the malaria vector system. However to date, most studies in Africa focused on *Anopheles gambiae s.l. *and, to a lesser extent, *Anopheles funestus *whereas research on other important vectors has critically lagged behind. Here, the first results of a large-scale population genetics analysis of the level and distribution of (neutral) genetic diversity in the mosquito *Anopheles nili *are presented.

*Anopheles nili *is a widespread efficient vector of *Plasmodium *parasites in the humid savannas and forested areas of sub-Saharan Africa [9-14]. It is the nominal taxon of a group of closely related species including *An. nili sensu stricto*, *Anopheles somalicus*, *Anopheles carnevalei *and *Anopheles ovengensis *[7,15]. The members of this group can be distinguished through slight morphologic diagnostic characters observable at the larval and/or adult stages [15,16] and a molecular diagnostic tool based on segregating sequence differences in the Internal Transcribed Spacer 2 (ITS2) of the ribosomal DNA (rDNA) [17]. Of these four species, *An. nili s.s. *(hereafter *An. nili*) is the most important malaria vector although *An. carnevalei *and *An. ovengensis *have been found infected with *Plasmodium falciparum *in natural conditions [9,15,18]. *Anopheles somalicus *is mainly zoophilic and is not involved in human malaria transmission [19,20]. Infection rates reaching 3% have been observed in *An. nili *and the species was shown to sustain entomological inoculation rates over 200 infected bites per man per year in villages close to fast running streams and rivers where its larvae develop [9-11,18]. Recent investigations of the ecological requirements of *An. nili *in Cameroon, a country in Central Africa at the core of the species range, showed that lotic rivers exposed to sunlight, with vegetation or debris were the best predictors of *An. nili *larval abundance [21] and that habitats characterized by high water vapor pressure and rainfall, as typically observed in forest-savanna transition areas were of highest quality for the development of the species [14]. *Anopheles nili *however is scarce in deep forest environments, where it is replaced by other members of the group, namely *An. carnevalei *and *An. ovengensis *[18,21]. The strong reliance of *An. nili *on permanent aquatic habitats for larval development suggests a patchy geographic distribution throughout the species' range, owing to the discontinuous nature of the hydrographic networks. This may lead to significant population genetic structure and behavioural differentiation. However, to date, very few studies have addressed the level and extent of genetic structuring within and between *An. nili *populations in sub-Saharan Africa. Sequence variation in two nuclear loci within the rDNA cluster (e.g., ITS2 and D3 domain in the 28S rDNA subunit, [17]) and isoenzymes [22] did not reveal any signature of genetic heterogeneity among *An. nili *populations collected throughout south Cameroon. However, the low polymorphism of the genetic markers used and the limited geographical scales covered by these studies precludes extrapolation of these findings outside of the study area and calls for further investigations.

Here, genetic polymorphism in *An. nili *was investigated and compared among collections from nine locations from throughout its distribution range in West and Central Africa using 11 recently described nuclear microsatellite DNA markers [23] and sequence variation in two nuclear (rDNA) and two mitochondrial (mtDNA) genes. It is shown that recently developed microsatellite markers are suitable tools to explore the population genetic structure of wild *An. nili *in Africa. All molecular markers suggested that *An. nili *populations from West Africa are genetically homogeneous with very low levels of genetic differentiation between them, whereas the rainforest domain in Central Africa might act as a geographical barrier to gene flow. Implications of these findings for vector control are discussed and areas for future research are highlighted.

## Methods

### Mosquito sampling and field processing of specimens

**Figure 1:**
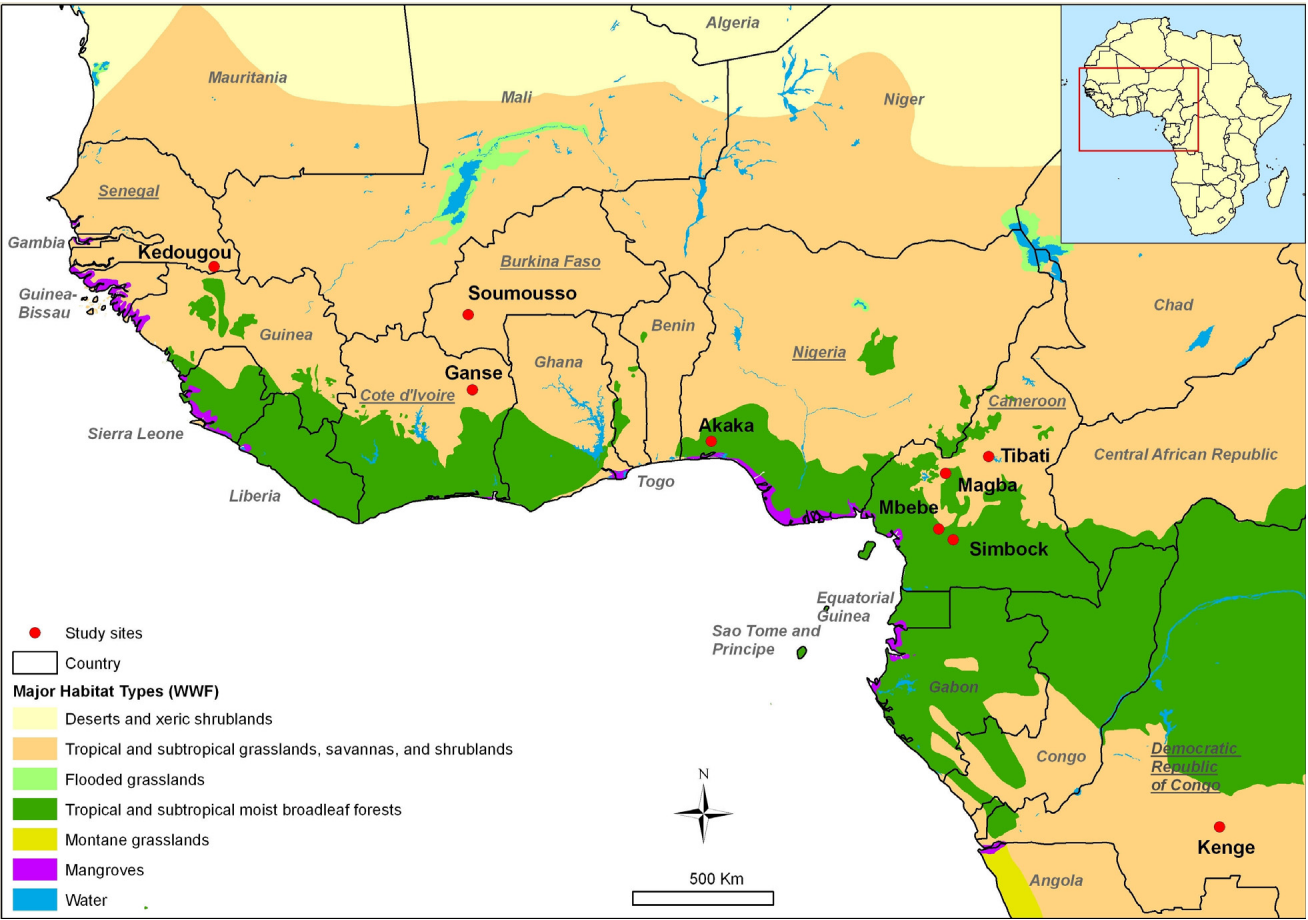
**Map of West and Central Africa showing collection sites**.

Mosquitoes were collected between June 2006 and March 2008 in nine localities across six countries from West and Central Africa (Figure 1), including Kedougou (12°38'N, 12°14'W) in Senegal, Soumousso (11°01'N, 4°03'W) in Burkina Faso, Gansé (8°37'N, 3°54'W) in Ivory Coast, Akaka (6°58'N, 3°44'E) in Nigeria, Simbock (3°49'N, 11°28'E), Mbébé (4°10'N, 11°04'E), Magba (5°57'N, 11°13'E) and Tibati (6°28'N, 12°36'E) in Cameroon and Kenge (5°19'S, 19°58'E) in the Democratic Republic of Congo (DRC). All these sites are located along different river systems in humid savanna areas except Simbock and Mbébé situated in the degraded forest area of southern Cameroon. The closest localities were Simbock and Mbébé situated 60 km apart, and the most distant ones were Kedougou and Kenge situated c.a. 3,000 km apart (Figure 1).

Anopheline female mosquitoes were collected by human landing catches and/or indoor pyrethrum spray catches. In the field, *An. nili s.l. *specimens were visually sorted from other anophelines according to morphological identification keys [19,24]. All specimens were stored individually in tubes containing a desiccant. All tubes were kept at -20°C until further analysis.

### DNA extraction and molecular identification

Genomic DNA was extracted from whole mosquito using a standard protocol described earlier [25]. Since distinction between members of the *An. nili *group is often difficult in the field because of very slight and stage-specific diagnostic differences between species, the molecular diagnostic PCR-based assay [17] was then performed to confirm morphological identification. Only *An. nili *mosquitoes were included in the analysis.

### Microsatellite genotyping and analysis

*Anopheles nili *mosquitoes were genotyped at eleven microsatellite loci (Table 1) [23]. All microsatellite loci are dinucleotide repeats. One primer of each pair was labeled with a 5' fluorescent dye (NED, PET, VIC or 6-FAM) to allow multiplex electrophoresis. PCR amplifications were carried out in 15 μL reaction volume from approximately 2 μL of template DNA (1/50 of the crude extract). The reaction mixture contained 1× PCR buffer (5 Prime Inc., Gaithersburg, USA), 1 mM MgCl_2_, 0.2 mM of each dNTP, 8 pmoles of each primer and 0.15 unit of Taq DNA Polymerase (5 Prime Inc.). Amplification runs were performed under the following conditions: an initial denaturation step at 94°C for 4 min followed by 35 cycles of 30 s at 94°C, 30 s at the appropriate annealing temperature (52°C to 56°C, see [23]) and 30 s at 68°C, followed by a final elongation step of 20 min at 68°C. PCR products were pooled with other compatible products according to allele size range and fluorescent dye. Mixtures were run on an ABI 3130XL DNA sequencer (Applied Biosystems, Foster City, USA) using 500 Liz internal size standard. Fragments (allele) sizes and genotypes were scored using GENEMAPPER V4.0 software (Applied Biosystems).

**Table 1:** Genetic variability at 11 microsatellite loci in nine *An. nili *populations from sub-Saharan Africa

	Senegal	B. Faso	Ivory C.	Nigeria	Cameroon				DRC	All
							
Locus	Kedougou	Soumousso	Gansé	Akaka	Tibati	Magba	Mbebe	Simbock	Kenge	samples
	(44)	(40)	(41)	(14)	(41)	(42)	(36)	(42)	(41)	(341)
1D80										
Rs	10.52	10.14	10.39	12.43	10.87	10.63	7.62	8.07	6.23	9.99
He	0.918	0.908	0.911	0.952	0.920	0.910	0.831	0.815	0.754	0.880
Fis	0.071	-0.014	0.043	0.048	0.214***	0.123*	-0.102	-0.007	0.072	0.077***
1A27										
Rs	12.80	12.42	13.17	13.41	12.70	10.93	11.47	11.36	6.98	12.39
He	0.946	0.943	0.953	0.953	0.949	0.926	0.930	0.930	0.831	0.929
Fis	0.281***	0.245***	0.229***	0.04	0.032	0.144**	0.049	0.182***	0.416***	0.204***
2Ateta										
Rs	9.10	9.71	9.03	9.62	9.33	8.49	9.29	8.77	4.55	9.00
He	0.895	0.901	0.876	0.879	0.875	0.866	0.896	0.879	0.395	0.829
Fis	-0.031	0.017	-0.028	-0.146	0.053	0.043	0.165*	0.03	-0.012	0.083***
A14										
Rs	5.87	6.63	6.60	4.31	6.34	6.92	5.38	5.47	1.61	5.84
He	0.695	0.745	0.737	0.499	0.732	0.772	0.655	0.675	0.077	0.621
Fis	0.318***	0.295***	0.036	-0.247	0.101	-0.052	-0.051	-0.017	-0.028	0.121***
A154										
Rs	8.46	8.92	9.25	8.17	9.88	8.79	9.10	7.48	8.41	9.68
He	0.796	0.830	0.837	0.841	0.854	0.788	0.778	0.736	0.800	0.807
Fis	0.406***	0.274***	0.194***	0.243***	0.252***	0.08	0.194***	0.016	-0.126*	0.203***
2C157										
Rs	5.55	4.83	4.82	5.99	4.70	5.32	5.47	5.73	2.25	5.04
He	0.728	0.707	0.657	0.651	0.713	0.743	0.702	0.778	0.505	0.687
Fis	0.074	0.058	-0.067	-0.102	-0.017	0.044	0.079	-0.049	0.211	0.046
F56										
Rs	9.89	9.33	10.56	10.06	10.06	9.61	10.02	7.80	4.56	9.36
He	0.904	0.890	0.918	0.900	0.903	0.898	0.903	0.847	0.561	0.858
Fis	0.07	0.059	0.08	-0.117	0.179***	0.054	0.116*	0.050	0.409***	0.131***
B115										
Rs	8.29	8.03	7.32	6.99	6.92	7.63	6.92	7.98	3.49	7.23
He	0.857	0.852	0.843	0.723	0.831	0.854	0.824	0.859	0.395	0.782
Fis	0.084	0.091	0.006	-0.179	-0.057	-0.021	0.227	-0.052	0.074	0.089***
F41										
Rs	12.53	12.96	12.83	12.41	12.49	12.42	12.54	12.62	8.47	12.62
He	0.945	0.947	0.947	0.942	0.944	0.943	0.943	0.944	0.755	0.923
Fis	0.028	-0.001	0.038	-0.064	0.023	-0.033	-0.061	-0.005	0.007	0.022
1F43										
Rs	6.25	7.16	7.12	4.00	7.06	6.37	7.27	7.52	8.50	7.35
He	0.768	0.793	0.787	0.768	0.836	0.821	0.832	0.846	0.874	0.814
Fis	0.023	0.209*	-0.052	-0.182	0.080	0.064	0.176***	0.115*	-0.008	0.081***
1G13										
Rs	6.75	7.54	6.18	6.33	6.82	6.36	6.06	6.27	4.68	6.45
He	0.796	0.827	0.773	0.743	0.790	0.784	0.753	0.780	0.657	0.767
Fis	0.125	0.142*	-0.057	-0.128	-0.068	-0.186	-0.063	0.031	-0.014	-0.008

All loci										
Rs	8.73	8.88	8.84	8.52	8.83	8.50	8.28	8.10	5.43	8.63
He	0.840	0.849	0.840	0.805	0.850	0.845	0.822	0.826	0.594	0.808
Fis	0.128***	0.122***	0.044	-0.063*	0.075***	0.026	0.068***	0.029	0.098***	0.097***

*Rs*: Allele richness; *He*: Nei's unbiased estimate of expected heterozygosity [28]. All loci/All samples: mean values across loci/populations; *P < 0.05, **P < 0.01 and ***P < 0.001 (single test level); In bold: significant (P < 0.05) heterozygote deficits according to exact tests against Hardy-Weinberg proportions after correction for multiple testing by the sequential Bonferroni procedure [39]; *Fis *was calculated according to [30]; In parenthesis: sample size. B. Faso: Burkina Faso, Ivory C.: Ivory Coast, DRC: Democratic Republic of Congo.

Genetic diversity by locus, within each geographical population and overall was assessed by estimates of allelic richness [26] and allelic frequencies using FSTAT V2.9.3 [27]. Allelic richness was used instead of the number of alleles per locus to account for differences in sample sizes. Estimates of expected heterozygosity under Hardy-Weinberg equilibrium [28] were obtained using GENETIX V4.02 [29].

Goodness-of-fit to Hardy-Weinberg equilibrium (HWE) for each locus and linkage disequilibrium between all pairs of loci were assessed using FSTAT, for each geographical population and overall. Whether deviations from HWE resulted from a deficit or an excess of heterozygotes was tested using *F*-statistics [30]. Significance tests were conducted using a randomization approach as implemented in FSTAT. The frequency of null alleles at each locus within each population was determined using GENEPOP V4.0 [31,32], and the allele and genotype frequencies were then adjusted accordingly in MICROCHECKER V2.2.3 [33]. The null allele adjusted dataset was compared to the original dataset to explore the effect of null alleles on estimations of genetic differentiation.

To investigate biased genetic differentiation caused by demographic instability such as bottlenecks and/or population expansion, heterozygosity tests were implemented to test for Mutation-Drift Equilibrium (MDE) using BOTTLENECK V1.0.02 [34]. BOTTLENECK compares two estimates of expected heterozygosity, one based on allele frequencies (*H*e) and the other based on the number of alleles and sample size (*H*eq). In a population at MDE, both estimates should not differ significantly (*H*e ≈ *H*eq). If a population experiences a bottleneck, rare alleles will be lost by genetic drift, and *H*eq will decrease faster than *H*e (*H*e > *H*eq). The resulting apparent excess of heterozygotes is an indicator of recent bottleneck event, whereas the opposite (*H*e <*H*eq) may signal an expansion process. Estimates of *H*eq were computed using two mutation models for microsatellites evolution: the Two-Phased Mutation model (TPM) [35] with fractions of multistep mutations set to 30%, 20% and 10%, and the Stepwise Mutation Model (SMM) [36]. Wilcoxon signed-rank tests were used to determine whether deviations from MDE were statistically significant.

Population genetic differentiation was measured by the fixation index Fst [30,37] and statistical significance was assessed by the exact test of genotypic differentiation available in FSTAT. Isolation by distance was investigated as a potential cause of genetic structuring using the Mantel test implemented in GENEPOP. The correlation between pairwise genetic differentiation and geographical distance was assessed by the regression of Fst/1-Fst on the logarithm of geographical distances between sampling sites [38]. The Bonferroni correction was used throughout to account for multiple testing [39].

Finally, a Bayesian clustering analysis was carried out using STRUCTURE V2.1 to cluster individuals into K groups while minimizing Hardy-Weinberg disequilibrium and gametic phase disequilibrium between loci within groups, with no a priori assumptions [40,41]. The software was run with the option of admixture, allowing for some mixed ancestry within individuals, and α was allowed to vary. Ten independent runs were done for each value of K (1 to 5), with a burn-in period of 100,000 iterations and 100,000 replications. The method implemented by Evanno *et al *[42] was used to estimate the most likely number of clusters in the dataset.

### DNA sequencing and analysis

Sequence variation was examined in the second Internal Transcribed Spacer (ITS2) and Domain-3 (D3) of nuclear 28S rDNA, and in the *Cytochrome oxidase subunit II *(COII) and the *NADH deshydrogenase subunit IV *(ND4) genes on mitochondrial DNA (mtDNA). Ten specimens per sites, among those used for microsatellite analysis, were randomly selected for sequencing. The ITS2, D3, COII and ND4 regions were amplified in 25 μL reaction mixtures containing 2.5 μL of 10× reaction buffer (QIAGEN, Courtaboeuf, France), 200 μM of each dNTP (Eurogentec, Angers, France), 0.5 unit of Taq DNA polymerase, and 10 pmol each of the forward and reverse primers. ITS2 and D3 rDNA regions were amplified using the primers sets ITS2a/ITS2b and D3a/D3b described in Kengne *et al *[17]. COII and ND4 were amplified using the following primers:

COIIF: 5'-TCTAATATGGGAGATTAGTGC-3' (Forward)

COIIR: 5'-ACTTGCTTTCAGTCATCTAATG-3' (Reverse)

ND4F: 5'-TGATTGCCTAAGGCTCATGT-3' (Forward)

ND4R: 5'-TTCGGCTTCCTAGTCGTTCAT-3' (Reverse)

The PCR conditions included an initial denaturation step at 94°C for 3 min, followed by 35 cycles at 94°C for 30 s, 55°C for 30 s, and 72°C for 45 s, with a final extension step at 72°C for 10 min. After DNA analysis by electrophoresis, PCR products were purified and used for sequencing in both directions with the previous primers, on an ABI 3130XL DNA sequencer (Applied Biosystems). Sequences were inspected and corrected, where necessary, using SEQSCAPE software (Applied Biosystems). Multiple sequence alignments for each gene were performed using MEGA V3.0 [43] and CLUSTALX [44]. Summary DNA sequence polymorphism statistics and measures of divergence were computed using DnaSP V4.10.9. [45]. Statistical tests of Tajima [46], Fu and Li [47] and Fu [48] also implemented in DnaSP were used to test for non neutral evolution and deviation from MDE. Phylogenetic relationships between *An. nili *haplotypes were inferred in NETWORK V4.5.1.0 software to create an unrooted haplotype network, using star contraction [49].

## Results

### Microsatellite analysis

#### Genetic diversity

Genotypes at 11 microsatellites loci were analysed in a total of 341 *An. nili *specimens originated from nine localities in six countries across West and Central Africa (Figure 1, Table 1). Individual genotypes are available upon request to the corresponding author. All loci amplified successfully and were highly polymorphic in all populations. Mean allelic richness (Rs) across populations ranged from 5.04 at locus 2C157 to 12.62 at locus F41, and average expected heterozygosity (He) across all samples ranged from 0.621 at locus A14 to 0.929 at locus 1A27 (Table 1). Populations from West Africa and Cameroon displayed very similar mean allelic richness (Rs = 8.10-8.88) and mean expected heterozygosity (He = 0.805-0.849) (ANOVA: P > 0.05). Both estimates were significantly lower (ANOVA: P < 0.05) in the Kenge population from DRC (Rs = 5.43, He = 0.594) (Table 1).

Departure from HWE (P < 0.05 after correction for multiple testing) were detected in 8 of 11 (72.72%) loci when all samples were pooled and considered as a single gene pool. All significant deviations were associated with a deficit in heterozygotes (positive values of Fis), suggesting population substructure (Table 1). When samples were split into geographical populations, 11 tests of 99 (11%) remained significant after the sequential Bonferroni procedure was applied. Three loci 1A27, A154 and A14 were particularly involved in nine of these deviations. No deviation was detected in three of the four samples from Cameroon (Magba, Mbebe and Simbock). The populations from Burkina Faso and Senegal with three deviations each had the highest number of loci out of HWE. All these deviations were attributed to the presence of null alleles, with frequency of unobserved alleles ranging from 0.088 to 0.184 in loci with significant heterozygote deficiency (see Additional file 1). However, null alleles did not significantly bias our interpretation, as re-analysis of adjusted datasets returned similar results.

Linkage disequilibrium (LD) tests were performed between all pairs of loci in all populations. Out of 495 comparisons, 7 (1.41%) were found significant at the single test level (P < 0.05): one in Gansé (1F43 × 1G13) and Kenge (A154 × F56), two in Simbock (1A27 × F41 and F56 × 1G13) and three in Tibati (2 Ateta × F56, A154 × B115 and 2C157 × 1G13). None of these tests remained significant after the sequential Bonferroni procedure was applied. These results suggest that loci segregate independently and confirm that null alleles are the most likely cause of the heterozygote deficiencies observed. Accordingly, all loci were included in the analysis and each geographical population was considered as a panmictic unit.

Heterozygosity tests were performed to explore demographic stability in *An. nili *populations and compliance to MDE. No signature of a recent bottleneck event was detected in any population. However, significant deviations (P < 0.01) from MDE which may suggest population expansion (He < Heq) were found in all populations under the SMM. In Kenge, deviations associated with strong heterozygote deficiencies were detected under all mutation models (Table 2).

**Table 2:** Heterozygosity tests in *An. nili *populations from sub-Saharan Africa.

Country	Locality		TPM		SMM
			
		70%	80%	90%	
Senegal	Kedougou	5	5	7	8**
B. Faso	Soumousso	5	5	9*	10**
Ivory C.	Gansé	6	6	6	9**
Nigeria	Akaka	7	7	7	9*
Cameroon	Tibati	4	5	5	9**
	Magba	5	5	6	9**
	Mbebe	5	5	6	9**
	Simbock	4	7	8	9**
DRC	Kenge	8**	9**	9**	10***

TPM: Two-Phase mutation Model with a % single step mutations, SMM: Stepwise Mutation Model; *P < 0.05, **P < 0.01 and ***P < 0.001 (two-tailed Wilcoxon signed-rank test P-values for deviation from MDE) after correction for multiple testing. B. Faso: Burkina Faso, Ivory C.: Ivory Coast, DRC: Democratic Republic of Congo.

The number of microsatellite loci showing heterozygote deficiency (e.g., He < Heq, see text) out of 11 test loci is given.

#### Population genetic structure and isolation by distance

**Figure 2:**
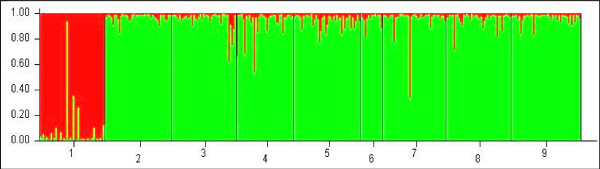
**Genetic cluster analysis using STRUCTURE based on multilocus microsatellites genotypes of *An. nili *specimens**. Graphical representation of the data set for the most likely number of genetic clusters (K = 2), where each color corresponds to a suggested cluster and each individual is represented by a vertical bar. The numbers in the X-axis correspond to a specific sample: 1-Kenge, 2-Magba, 3-Tibati, 4-Mbebe, 5-Simbock, 6- Akaka, 7-Soumousso, 8-Gansé and 9-Kedougou. The Y-axis represents the probability of assignment of an individual to each cluster.

A Bayesian clustering analysis performed with the software STRUCTURE revealed that the most likely number of genetic clusters in the dataset is 2. The first cluster included the sample from Kenge (DRC) and the second cluster grouped together all other samples (Figure 2). Clustering analysis was repeated without the Kenge population to examine whether any further sub-structuring was present within the second cluster and did not detect any additional sub-structuring.

All populations displayed similar allele profiles, with a lack of rare alleles in the *An. nili *population from Kenge, which appeared highly differentiated from the rest (Table 3). High Fst estimates (Fst = 0.119-0.153, P < 0.001) were observed between this population and all the other ones. In contrast, low genetic differentiation (at least one order of magnitude lower) was detected among the remaining populations (i.e., within cluster 2 identified above). To verify whether genetic differentiation between Kenge and the rest was specific to particular loci, jackknifed Fst estimates were calculated after excluding one locus at a time. This analysis provided consistent results reflecting homogeneity of Fst estimates across the set of loci (Table 4).

**Table 3:** Pairwise Fst estimates between *An. nili *populations from sub-Saharan Africa using 11 microsatellite loci.

		Senegal	B. Faso	Ivory C	Cameroon					DRC
						
		Kedougou	Soumousso	Gansé	Akaka	Tibati	Magba	Mbebe	Simbock	Kenge
Senegal	Kedougou	-								
B. Faso	Soumousso	0.0000	-							
Ivory C.	Gansé	0.0017	0.0000	-						
Nigeria	Akaka	0.0111	0.0107*	0.0058	-					
Cameroon	Tibati	0.0018*	0.0014***	0.0000	0.0031	-				
	Magba	0.0024*	0.0058***	0.0025*	0.0073*	0.0000	-			
	Mbebe	0.006***	0.0075***	0.004*	0.0133***	0.0013	0.0037	-		
	Simbock	0.0091***	0.0128***	0.0114***	0.0222***	0.0063***	0.0049***	0.0000	-	
DRC	Kenge	0.1251***	0.1328***	0.124***	0.1531***	0.1242***	0.1356***	0.1275***	0.1189***	-

*P < 0.05, **P < 0.01, ***P < 0.001. In bold: significant Fst values after corrections for multiple testing using the sequential Bonferroni procedure. B. Faso, Burkina Faso; Ivory C., Ivory Coast; DRC, Democratic Republic of Congo.

**Table 4:** Jackknifing over loci for the estimation of overall genetic differentiation between the two *An. nili *clusters.

Locus removed	Fst	P-value
1A27	0.121	P < 0.0001
1D80	0.121	P < 0.0001
1F43	0.118	P < 0.0001
1G13	0.120	P < 0.0001
2Ateta	0.101	P < 0.0001
2C157	0.120	P < 0.0001
A14	0.110	P < 0.0001
A154	0.108	P < 0.0001
B115	0.102	P < 0.0001
F41	0.117	P < 0.0001
F56	0.112	P < 0.0001

None	0.114	P < 0.0001

**Figure 3:**
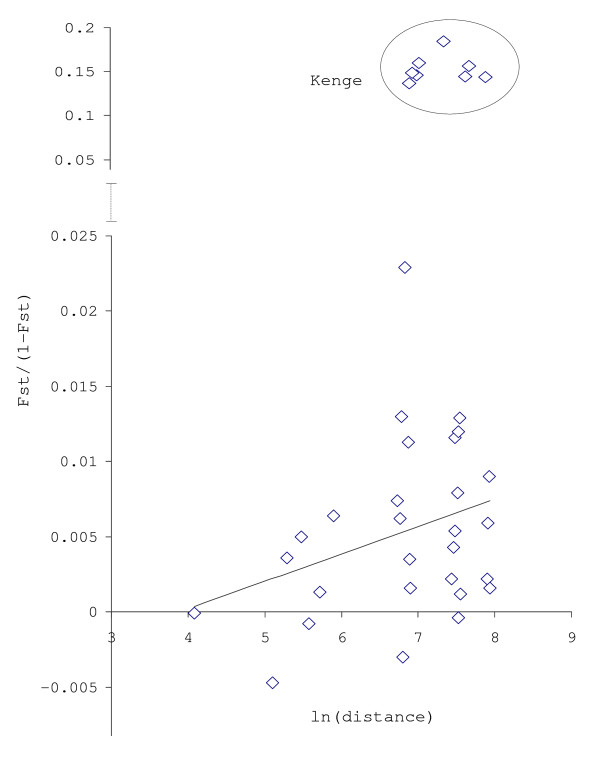
Correlation between average Fst estimates and logarithm of geographic distance between collection sites for pairwise comparisons of *An. nili *populations from sub-Saharan Africa

Because geographical isolation is often the main force driving population differentiation, the level of genetic differentiation which could be attributable to the geographical distance between collection sites was assessed using Mantel tests. A positive and significant correlation was found between Fst/(1-Fst) and logarithm of geographic distance (Mantel test: P < 0.05) when all samples were included in the analysis (Figure 3), suggesting that geographic distance between sites is responsible for part of the differentiation observed. Nevertheless, the correlation was not significant (Mantel test, P > 0.05) when Kenge was excluded from the analysis.

### Sequence analysis

#### **rDNA polymorphism**

In total, 76 sequences were generated for ITS2 and D3 each. These sequences perfectly matched those published by Kengne et *al*. [17]. Of the 451 bp of ITS2, no variable sites were found, whereas only one variable site (mutation) was found in one specimen in Kenge (DRC) among the 392 pb of the D3 domain (GenBank Accession Number:GU947798). These results are consistent with the fact that all *An. nili *populations analysed belonged to the same taxonomic unit.

#### mtDNA polymorphism and divergence

**Figure 4:**
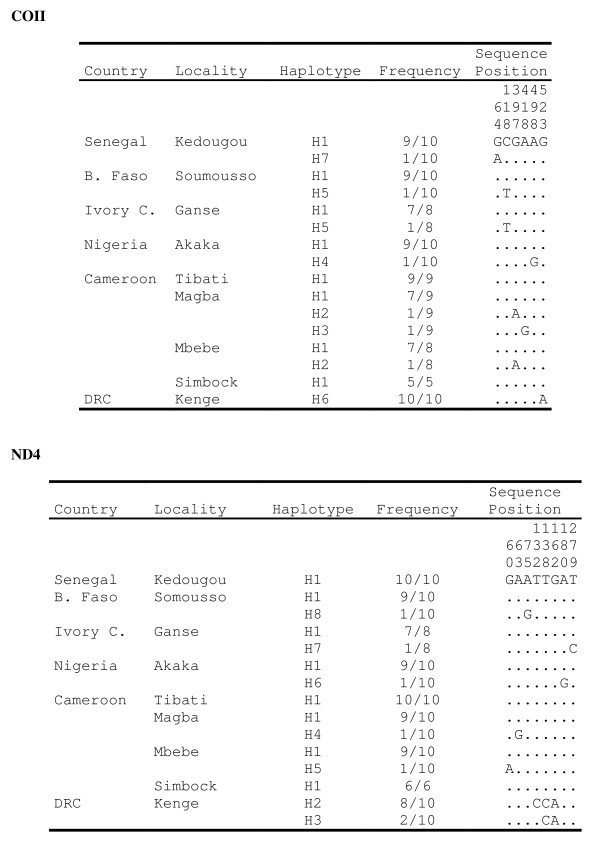
Frequency and distribution of haplotypes in COII (above) and ND4 (below) genes in nine *An. nili *populations

The nucleotide sequence was determined along 603 bp of the COII gene (coding region) in a total of 78 individual mosquitoes. In addition, a fragment of 320 bp within the coding region of ND4 gene was obtained for 84 mosquitoes. All segregating sites and the sequence variants (haplotypes) are shown in Figure 4 and summary statistics for both genes are given in Table 5.

**Table 5:** Summary statistics for mtDNA genes polymorphism and neutrality tests in *An. nili *from sub-Saharan Africa

	Senegal	B. Faso	Ivory C	Nigeria	Cameroon				DRC	All
							
Gene	Kedougou	Soumousso	Gansé	Akaka	Tibati	Magba	Mbebe	Simbock	Kenge	samples
COII										
n	10	7	8	10	9	9	8	5	10	76
s	1	1	1	1	0	2	1	0	0	6
hd	0.200	0.286	0.250	0.200	0.000	0.417	0.250	0.000	0.000	0.383
π	0.0003	0.0005	0.0004	0.0003	0.0000	0.0007	0.0004	0.0000	0.0000	0.0007
*D*_*T*_	-1.111	-1.006	-1.055	-1.111	nc	-1.362	-1.055	nc	nc	-1.564
*D*	-1.243	-1.048	-1.126	-1.243	nc	-1.505	-1.126	nc	nc	-1.632
*F*	-1.347	-1.101	-1.203	-1.347	nc	-1.626	-1.203	nc	nc	-1.896
*Fs*	-0.339	-0.095	-0.182	-0.339	nc	-1.081	-0.182	nc	nc	-4.999**
ND4										
n	10	10	8	10	10	10	10	6	10	84
s	1	1	1	1	0	1	1	0	1	8
hd	0.200	0.200	0.250	0.200	0.000	0.200	0.200	0.000	0.200	0.338
*D*_*T*_	-1.111	-1.111	-1.055	-1.111	nc	-1.111	-1.111	nc	-0.111	-1.302
*D*	-1.243	-1.243	-1.126	-1.243	nc	-1.243	-1.243	nc	-1.243	-1.914
*F*	-1347	-1347	-1.205	-1347	nc	-1347	-1347	nc	-1347	-2.100
*Fs*	-0.339	-0.339	-0.181	-0.339	nc	-0.339	-0.339	nc	0.356	-3.414*
π	0.0006	0.0006	0.0008	0.0006	0.0000	0.0006	0.0006	0.0000	0.0010	0.0023

n: number of sequences; s: number of polymorphic sites; hd: haplotype diversity; π : nucleotide diversity; *D*_*T*_: Tajima's *D*; *D*: Fu and Li's *D*; *F*: Fu and Li's F; *Fs*: Fu's *Fs *statistics; nc : not computed; *: P < 0.05; **: P < 0.01; B. Faso: Burkina Faso, Ivory C.: Ivory Coast, DRC: Democratic Republic of Congo.

Overall, low polymorphism was found in both mitochondrial genes. Across the whole dataset, there were only 6 (0.99%) and 8 (2.5%) polymorphic sites for COII and ND4, respectively. This low number of variable sites resulted in low nucleotide diversity (π < 0.001 in all samples, Table 5) and low haplotype diversity (hd < 0.42, Table 5) across samples. Among the 78 COII sequences, seven haplotypes (overall hd = 0.375) were found and three of them appeared in a single copy (singleton) in the dataset. The most frequent haplotype was identical to the reference sequence published by Marshall *et al *[50] (GenBank Accession Number: DQ069720). GenBank Accession numbers for the other 6 COII haplotypes are GU947799 to GU947804. Similarly to COII gene, 4 of 8 ND4 haplotypes (overall hd = 0.331, GenBank Accession Numbers: GU947805 to GU947812) were singletons. Tajima's *D*_*T *_, Fu & Li's *F *and *D*, and Fu's *Fs *statistics were all negative for both genes, although only *Fs *reached statistical significance when all samples were pooled (Table 5). Negative values of these statistics might reflect either a selective sweep or a recent demographic expansion [48], a finding that is in agreement with data obtained from the microsatellite analysis.

**Figure 5:**
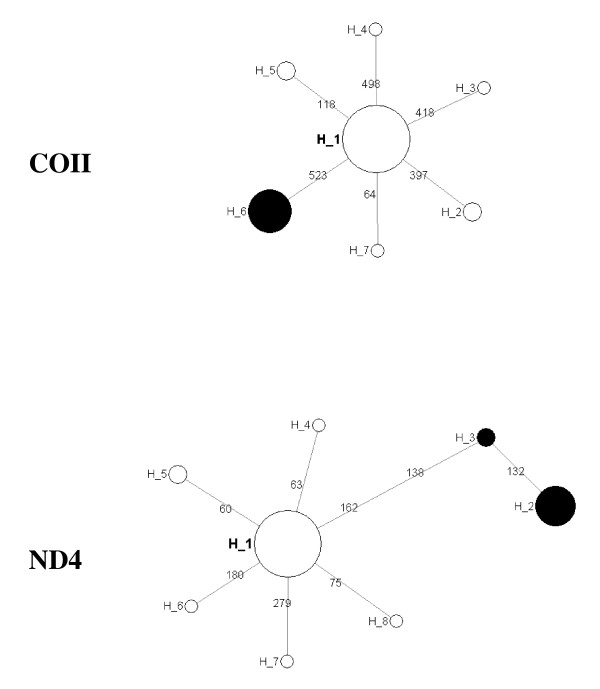
**Haplotype networks for COII (above) and ND4 (below) genes in *An. nili***. Numbers are the mutation positions on the reference sequence. Circles are roughly proportional to haplotype frequencies. Black circles identify haplotypes unique to Kenge population (GeneBank Accession Numbers: GU947803 for COII; GU947806-GU947807 for ND4).

For either gene, eight of nine populations (excluding Kenge) shared their most frequent haplotype and the frequency of this haplotype ranged from 78% to 90% (Figure 4). No haplotype was shared between Kenge and the other samples. The sequences of specimens from Kenge differed from the rest by one and two fixed mutations in COII and ND4, respectively (Figure 4). Graphical analysis of the genealogical relationships among all mtDNA sequences using haplotype networks showed that all *An. nili *haplotypes derived from a single common ancestral haplotype (Figure 5). This star-like network of haplotype again suggested recent population expansion.

## Discussion

This is the first study using microsatellite markers to explore *An. nili *population genetic structure. All loci successfully amplified in each population and were highly polymorphic compared to the isoenzyme markers previously used [22]. This is consistent with previous studies comparing microsatellite to isoenzymes markers in anophelines species [e.g., [51-53]]. Likewise, microsatellite loci were more polymorphic than the rDNA and the mtDNA genes used in this study. This pattern may reflect the small sample sizes used for sequencing and/or insufficient resolution of the molecular markers [54]. Moreover, a biological process such as different evolutionary rates of the markers or locus/region-specific selective constraints could also be involved [55,56]. Nonetheless, despite this heterogeneity in overall polymorphism across molecular markers, the same trends emerged whereby *An. nili *populations from West and Central Africa (i.e., from Senegal to Cameroon) appeared genetically homogeneous, whereas mosquitoes sampled in DRC were highly differentiated from the species' core populations. In addition, although individual inferences were rather weak, all markers showed a pattern of diversity and distribution of molecular polymorphisms that is consistent with recent demographic expansion of *An. nili *throughout its distribution range in West/Central Africa.

Genetic homogeneity within the rDNA genes is not surprising, given the particular evolutionary dynamics of the rDNA operon subject to concerted evolution [e.g. [57]], rendering it extremely useful for the resolution of deep phylogenies and/or to distinguish between cryptic species but conversely, of little use for within-species population genetics analysis [55,58]. Mitochondrial DNA evolves faster than the nuclear genome and has been widely used for population genetics and phylogenetics, including arthropod vectors of human diseases [54,55,59]. Because of strongly biased AT content, this non-recombining molecule is however subject to saturation, leading to a rapid lost of phylogenetic signal through homoplasy [55,59]. On the other hand, its lower effective population size (1/4th that of nuclear markers due to maternal inheritance and haploidy) together with increased selection against slightly deleterious mutations [60] can rapidly increase divergence between lineages within species and reduce local genetic diversity due to enhanced genetic drift and/or molecular hitchhiking resulting in selective sweep throughout the mitochondrial genome [61,62]. This is in agreement with increased differentiation of the *An. nili *population from DRC observed with both mtDNA genes. Apparent segregation of different haplotypes between these two genetic clusters in *An. nili *prompts for further investigation to increase sample sizes and the geographic span of sampling eastwards and southwards of the present study area.

Reduced variability and increased differentiation of the DRC population was also detected using nuclear DNA microsatellite markers. Within each geographical population, HWE was generally respected. Evidence for null alleles at certain loci, as formerly observed by Berthomieu *et al *[23], did not obscure the pattern of differentiation between populations, suggesting the set of loci used in this study were able to capture the main patterns of genetic variability within the dataset. Extensive allele sharing between populations and homogeneity across loci in the level of genetic differentiation suggests enhanced genetic drift in the DRC population, rather than selection was responsible of the pattern observed. Unfortunately, the chromosomal locations of the markers remain unknown in the absence of a reliable chromosomal map for *An. nili *but linkage equilibrium between markers suggests that they are, at least statistically independent and the results might reflect a genome-wide pattern. Reduced variability and increased differentiation is typically observed in populations leaving in marginal habitats at the edge of species' ranges [63] and the sparsely populated evergreen forest block of Central Africa is known to be of low overall quality for the development of *An. nili *which is more frequent at the savanna/forest ecotone [14,21]. Deviation from MDE observed under a range of mutation models in this isolated population indeed suggests unstable demography, although no evidence for a recent bottleneck was obtained. All molecular markers suggested recent demographic expansion in the Kenge and, to a lesser extent, in all other *An. nili *populations sampled. Detection of this pattern in multiple independent loci make it possible to distinguish it from the effect of selection, which is locus-specific, and attribute it to past demographic change. This is reminiscent of the situation observed in other major vectors of human malaria in Africa and elsewhere [64], and prompts for further studies to disentangle the confounding effect of shared ancestral polymorphism from that of ongoing gene flow between geographical populations [59]. Moreover, the role of the evergreen forest block as a geographic barrier to gene flow between *An. nili *populations needs to be further explored, given the low dispersal ability of this mosquito in this environment [65]. Clearly, extending the sampling area eastwards and southwards is needed to provide an overall picture of the level and distribution of genetic diversity within *An. nili *throughout its distribution range on the continent, and identify both geographic barriers that prevent gene flow between populations and areas of extensive gene exchange as seems to be the case throughout West Africa. Such knowledge is needed to devise efficient, locally adapted and sustainable strategies for the management and control of these vector populations. It is interesting to note that recent investigations of the population genetic structure of *An. moucheti *sampled in the same localities as presented here (at least in Cameroon and the DRC) did not detect such high level of population differentiation within and across the forest block [53,66]. Combined analysis of genetic and ecological data in a comparative framework should reveal further insights into the population biology and demographic history of these neglected malaria vectors, and provide relevant information for their control. Recent advances in theoretical population genetics and the rapidly evolving field of spatial genetics [e.g., [56]] together with the development and democratization of high throughput sequencing technologies provide the necessary tools for such endeavor in non model species.

## Competing interests

The authors declare that they have no competing interests.

## Authors' contributions

CN, CAN, IM and FS designed the study and monitored its implementation. CN, CAN, PAA participated to field sampling. CN conducted microsatellite genotyping and gene sequencing, under guidance of PK and IM. CN, PK, DA, AC and FS participated to data analysis. CN, CAN and FS wrote the manuscript which was critically revised by AC, DA, PN and DF. DC was involved in the design of the maps contained in this paper. All authors read and approved the final manuscript.

## Supplementary Material

Additional file 1 **Supplementary table S1**. Estimates of null alleles frequencies per locus and per geographical population of *An. nili*. Click here for file
