# Supplementary material for: Population genetic structure of the malaria vector Anopheles nili in sub-Saharan Africa
Source: Malar J. 2010 Jun 12;9:161. doi: 10.1186/1475-2875-9-161 (PMC2898787; doi:10.1186/1475-2875-9-161)
Supplement: Additional file 1 — Supplementary table S1. Estimates of null alleles frequencies per locus and per geographical population of An. nili. [file 1475-2875-9-161-S1.DOC]

**Supplementary table S1:** Estimates of null alleles frequencies per locus and per geographical population of *An. nili*.

|  | Senegal | B. Faso | Ivory C. | Nigeria |  | Cameroon | | | |  | DRC |
| --- | --- | --- | --- | --- | --- | --- | --- | --- | --- | --- | --- |
|  | Kedougou | Soumousso | Gansé | Akaka |  | Tibati | Magba | Mbebe | Simbock |  | Kenge |
| 1D80 | 0.031 | 0.000 | 0.005 | 0.048 |  | **0.089** | 0.044 | 0.000 | 0.000 |  | 0.000 |
| 1A27 | **0.153** | **0.115** | **0.109** | 0.000 |  | 0.009 | 0.057 | 0.002 | 0.079 |  | **0.184** |
| 2Ateta | 0.000 | 0.017 | 0.000 | 0.000 |  | 0.000 | 0.026 | 0.071 | 0.000 |  | 0.000 |
| A14 | **0.097** | **0.125** | 0.000 | 0.000 |  | 0.022 | 0.011 | 0.000 | 0.015 |  | 0.000 |
| A154 | **0.166** | **0.088** | 0.102 | 0.078 |  | **0.088** | 0.052 | 0.048 | 0.000 |  | 0.000 |
| 2C157 | 0.000 | 0.009 | 0.000 | 0.000 |  | 0.000 | 0.000 | 0.005 | 0.000 |  | 0.065 |
| F56 | 0.017 | 0.019 | 0.029 | 0.000 |  | 0.089 | 0.000 | 0.059 | 0.000 |  | **0.168** |
| B115 | 0.036 | 0.010 | 0.002 | 0.000 |  | 0.000 | 0.000 | 0.071 | 0.000 |  | 0.008 |
| F41 | 0.007 | 0.000 | 0.003 | 0.000 |  | 0.008 | 0.000 | 0.000 | 0.000 |  | 0.000 |
| 1F43 | 0.000 | 0.090 | 0.000 | 0.000 |  | 0.050 | 0.000 | 0.099 | 0.090 |  | 0.011 |
| 1G13 | 0.013 | 0.061 | 0.015 | 0.000 |  | 0.000 | 0.000 | 0.000 | 0.000 |  | 0.000 |

Bolded : loci showing significant (P<0.05) heterozygote deficiency after correction for multiple testing (bolded *Fis* values in Table 1).
